# Amyloid Imaging and *APOE* Genotype Disclosure and Short-Term Psychological Distress

**DOI:** 10.1001/jamanetworkopen.2026.3845

**Published:** 2026-03-30

**Authors:** Joshua D. Grill, Rema Raman, Shunran Wang, Karin Ernstrom, Patricia S. Andrews, Brian S. Appleby, Jaspreet Bhangu, Shobha Dhadda, Michael Irizarry, Keith Johnson, Steven Lenio, Steven MacDonald, Vijay K. Ramanan, Paul B. Rosenberg, David Weisman, Paul Aisen, Reisa Sperling, David Sultzer, Jason Karlawish

**Affiliations:** 1 Institute for Memory Impairments and Neurological Disorders, University of California, Irvine; 2 Epstein Family Alzheimer's Therapeutic Research Institute, University of Southern California, San Diego; 3 Department of Psychiatry and Behavioral Sciences, Vanderbilt University Medical Center, Nashville, Tennessee; 4 Departments of Neurology, Psychiatry, and Pathology, Case Western Reserve University School of Medicine, Cleveland, Ohio; 5 University Hospitals Cleveland Medical Center, Cleveland, Ohio; 6 St Joseph’s Health Care London, London, United Kingdom; 7 Eisai, Nutley, New Jersey; 8 Center for Alzheimers, Harvard, Brigham and Women’s Hospital, Boston, Massachusetts; 9 Department of Neurology, Boston University, Boston, Massachusetts; 10 Department of Neurology, University of Michigan, Ann Arbor; 11 Department of Neurology, Mayo Clinic, Rochester, Minnesota; 12 Department of Psychology, Johns Hopkins University, Baltimore, Maryland; 13 Abington Neurologic Associates, Abington, Pennsylvania; 14 University of Pennsylvania, Philadelphia

## Abstract

**Importance:**

Alzheimer disease (AD) biomarker and genetic testing results are increasingly disclosed to cognitively unimpaired adults in research and could in the future inform clinical treatment decisions in this population.

**Objectives:**

To assess psychological outcomes after returning 3 categories of amyloid biomarker results as well as apolipoprotein E (*APOE*) genotypes.

**Design, Setting, and Participants:**

This cohort study was a secondary analysis of data collected as part of screening for the multisite AHEAD preclinical AD trial. Participants were individuals aged 55 to 80 years undergoing screening from July 14, 2020, to October 15, 2024.

**Exposure:**

Participants were informed whether they had not-detected, intermediate, or elevated amyloid positron emission tomography levels, as well as their *APOE* genotype, which were categorized as noncarrier, ε4 heterozygote, or ε4 homozygote.

**Main outcomes and measures:**

Impact of Events Scale (IES; 15 items to assess intrusive thoughts and avoidance; each item is scored as not at all [0], rarely [1], sometimes [3], or often [5]; total range, 0-75), collected 24 to 72 hours after disclosure, and change in a scale measuring concerns about AD dementia (adapted scale using 6 items in which participants indicated their level of agreement with statements related to their perceived probability of developing AD dementia; items scored as strongly disagree [1] through strongly agree [5]; total range, 6-30), calculated by subtracting the score collected before biomarker testing from 1 collected after biomarker and genetic test results disclosure.

**Results:**

Among 3414 included individuals, the mean (SD) age was 68.8 (6.0) years and 2116 (62%) were female. Group mean IES scores were below clinically significant thresholds. Nevertheless, across genetic groups, learning an elevated amyloid result (1184 participants) was associated with higher IES (mean [SD], 10.5 [10.9]) than intermediate amyloid (482 participants; mean [SD] IES, 8.8 [9.8]), and intermediate amyloid was associated with higher scores than not-detected amyloid (1748 participants; mean [SD] IES, 6.5 [8.4]). Across amyloid groups, learning *APOE* ε4 homozygosity (337 participants) was associated with higher mean (SD) IES (12.7 [11.6]) than heterozygosity (1609 participants; 9.1 [10.2]), and heterozygosity was associated with higher IES than noncarrier status (1468 participants; mean [SD] IES, 6.2 [8.1]). Both types of information were significant in an analysis of covariance model; no interaction effect was observed. In contrast, only biomarker disclosure was associated with differential change in concerns about AD dementia. Those with elevated amyloid showed a mean (SD) increase in concern (0.8 [3.5]), those with intermediate amyloid showed a smaller increase (0.4 [3.7]), and those with not-detected amyloid showed decreased concerns (−1.1 [4.2]).

**Conclusions and Relevance:**

In this cohort study of cognitively unimpaired adults, associations with intrusive thoughts were observed to differ among genetic and biomarker subgroups; such associations were limited to biomarker subgroups for measures of perceived dementia risk.

## Key Points

**Question:**

Do psychological outcomes differ among cognitively unimpaired research participants aged 55 to 80 years who were disclosed 3 categorical outcomes of Alzheimer disease (AD) amyloid biomarker results as well as apolipoprotein genotypes?

**Findings:**

In this cohort study of 3414 individuals, both types of disclosure were associated with intrusive thoughts in a biomarker and gene dose association—greater risk categories were associated with higher scores on the Impact of Events Scale. In contrast, only biomarker results were associated with a change in the level of concern about developing AD dementia.

**Meaning:**

These findings suggest that biomarker and genetic information had independent associations with psychological outcomes in cognitively unimpaired adults.

## Teaser

This cohort study evaluates psychological outcomes after returning 3 categories of amyloid biomarker results as well as apolipoprotein E (*APOE*) genotypes among adults screened for participation in a randomized clinical trial.

## Introduction

New diagnostic criteria for Alzheimer disease (AD) posit that in the absence of symptoms, positive biomarker results are sufficient for diagnosis.^1^ Alternative criteria posit this same combination of features as a risk category.^2^ Despite these differences, both approaches agree: delivery of biomarker information to cognitively unimpaired individuals should not be performed until rigorous clinical trials demonstrate safe and effective treatments that delay the onset of cognitive impairment.

Ongoing trials are testing antiamyloid monoclonal antibody (AAMA) treatments in preclinical stages; that is, in individuals with biomarker evidence of disease but no overt cognitive impairment.^3^ One of these, the AHEAD Study, includes 2 trials, the A45 and the A3 trials, assessing the safety and efficacy of the AAMA lecanemab.^4^ The A45 trial is similar to previous preclinical AD trials in that it includes individuals with elevated brain amyloid (defined as >40 centiloids [CL] on amyloid positron emission tomography [PET] imaging). The A3 study enrolls individuals with intermediate amyloid, a category based on lower levels of brain amyloid (20-40 CL) and hypothesized to reflect an even earlier biological phase of disease.^5,6^

Individuals screened for AHEAD underwent PET imaging and then were told whether they had not-detected, intermediate, or elevated brain amyloid. Given the observed AAMAs’ risk of microscopic brain bleeds and swelling based on a patient’s apolipoprotein E (*APOE*) genotype,^7,8,9^ participants were also given the opportunity to learn their *APOE* genotype, which is associated with risk of AD dementia but is neither necessary nor sufficient for that outcome. Alone and together, these protocol elements present the opportunity to assess important psychological outcomes after learning AD biomarker and genetic risk information.

The objective of this study was to assess short-term psychological outcomes after returning 3 categories of amyloid biomarker results as well as *APOE* genotypes to cognitively unimpaired adults. Given that education on the meaning of these 2 types of information was provided as an element of consent, counseling, and disclosure in the trial, we hypothesized that dose responses would be observed among subgroups based on specific biomarker and genetic results on measures of intrusive thoughts and concerns about AD dementia. The results may inform a future clinical practice when clinicians diagnose and treat individuals with preclinical AD.

## Methods

### Data Source and Design

The AHEAD Study (clinicaltrials.gov NCT04468659) is a public-private partnership, funded by the National Institute on Aging, Eisai, and other nonprofit and philanthropic sources. Two separate trials, the A45 trial and A3 trial, used a shared recruitment, enrollment, and screening process, although individuals were randomized into the 2 separate protocols based on their screening biomarker results.^4^

The AHEAD Study was approved by the Advarra central institutional review board. This article describes only deidentified data and therefore does not meet the criteria for human participants research, so it was exempt from review and the need for informed consent in accordance with 45 CFR §46. Given that we report observational results, rather than outcomes of either trial, we adhered to the Strengthening the Reporting of Observational Studies in Epidemiology (STROBE) reporting guidelines.

### Participants

Screening occurred July 14, 2020, to October 15, 2024. To be eligible to screen for AHEAD, participants were required to be between the ages of 55 and 80 years, cognitively unimpaired (eg, no diagnosis of mild cognitive impairment or dementia), and otherwise healthy. Individuals aged 55 to 59 years were required to have 1 additional risk factor for AD to enroll, such as immediate family history of disease, subjective memory complaints, or a known *APOE* ε4 carrier status. Participants self-reported race and ethnicity and other demographic variables. Race and ethnicity were assessed in this study to assess sample diversity for eventual use in examining treatment effects. Participants were required to undergo amyloid PET imaging and to learn their results. Participants also had the option to learn their *APOE* genotype. For a brief period, *APOE* genotyping was delayed and not available to participants during screening. These and any other individuals not learning their *APOE* genotype during the screening period (699 participants among those who underwent amyloid PET imaging) had the option to learn their *APOE* genotype later or to delay enrolling until such time as their *APOE* genotype was available. eTable in Supplement 1 compares these individuals with individuals included in this analysis.

### Disclosure

All individuals screened for the trial who underwent amyloid PET imaging were informed of their biomarker result through a protocolized process, adapted from previous versions that disclosed elevated and not elevated results^10,11^ to include a third category of intermediate amyloid (defined in AHEAD as 20-40 CL). Individuals with elevated amyloid were informed that amyloid plaques were present in their brain, that this put them at increased risk for developing cognitive impairment and dementia, and that this made them eligible for the A45 trial. Individuals disclosed an intermediate amyloid result were informed that they had greater signal on their amyloid PET scan than individuals who had not-detected amyloid (<20 CL), but not at the level of being elevated. They were told that their risk of developing cognitive impairment was higher than if their scan result had been not detected but not as high as if their scan result had been elevated. They were informed that they were at increased risk to someday develop elevated amyloid and that this made them eligible to participate in the A3 trial. For both intermediate and elevated amyloid, participants were informed that their result did not mean that they would definitely develop AD dementia.

Participants had the option to learn their *APOE* genotypes. As with amyloid disclosure, a process was implemented that began with education and informed consent. Participants were informed about the nature of genetic inheritance, common *APOE* alleles (ε2, ε3, and ε4), and the 6 potential *APOE* genotypes. Educational materials emphasized that an individual can have no copies of ε4 and still develop AD dementia and that a person can have 1 or 2 copies of ε4 and not develop dementia. The materials also described the risk association for ε4 carriers for the main treatment-related adverse events for lecanemab, amyloid related imaging abnormalities of the edema, and hemorrhagic types.^8^ Study participant materials also addressed the safety of disclosure, legal risks (including lack of legal protection for some risks), implications of genetic testing to family members, confidentiality, and the role of the study partner in the AHEAD Study.

Biomarker and genetic test results were returned in person at the same visit by a site investigator who had undergone training and self-certification to deliver sensitive results to participants. This included confirmation of reading and adhering to a specific amyloid and *APOE* disclosure chapter in the study procedures manual. Participants were encouraged to bring their study partner to the disclosure visit.

After completing approximately one-third of study enrollment, a protocol amendment was introduced to use plasma biomarker testing to enrich the sample proceeding to PET screening and reduce the number of ineligible PET scans.^12^ After the implementation of this amendment, participants were informed whether their plasma biomarker test result made them eligible to continue screening. No other changes were implemented to the disclosure or data collection procedures.

### Outcomes

This retrospective analysis included data from 2 outcome measures collected during the screening and disclosure process. The Impact of Events Scale (IES)^13^ is a measure of subjective distress composed of items rating intrusive thoughts and avoidance previously incorporated in a variety of studies assessing the psychological impact of biomarker and genetic testing disclosure.^11,14,15^ The IES uses 15 items to assess intrusive thoughts and avoidance (eg, “I thought about it when I didn’t mean to”). Each item is scored as not at all (0), rarely (1), sometimes (3), or often (5), for a total range of 0 to 75. The IES used in this study referred to the participant receiving their amyloid PET result as the specific event, although amyloid biomarker and *APOE* results were returned at the same visit. Site personnel collected the IES during telephone follow-up with participants 24 to 72 hours after the in-person disclosure visit. The IES has been widely used for assessing the psychological outcomes of stressful events including trauma and thus is applicable to the potentially stressful event of learning one’s biomarker and genetic status.

We also examined data for an adapted scale measuring concerns about AD dementia.^16,17^ The adapted scale used 6 items in which participants indicated their level of agreement (strongly disagree, scored as 1, through strongly agree, scored as 5; total range: 6-30) with statements related to their perceived probability of developing AD dementia (eg, “I believe I will someday develop Alzheimer‘s disease dementia”) and their level of concern for that outcome (eg, “My concern about developing Alzheimer‘s disease dementia is greater than my concern about other medical problems”). The scale was collected at an initial screening visit and after in-person disclosure of biomarker and *APOE* results. Change was calculated by subtracting the score collected before biomarker testing from 1 collected after biomarker and genetic test results disclosure.

### Statistical Analysis

We restricted these analyses to individuals undergoing screening for the AHEAD study who (1) underwent amyloid PET imaging and (2) opted in and were able to learn their *APOE* status at the same disclosure visit. Participant characteristics were summarized with frequencies and percentages for categorical variables, and with means and SDs for continuous data. We assessed differences in these outcomes among 9 groups based on amyloid status (not detected, intermediate, and elevated) and *APOE* ε4 carrier status (noncarrier, heterozygote, or homozygote). We hypothesized that both biomarker and genetic information would be associated with psychological outcomes, such that differential responses would be observed among subgroups based on specific biomarker and genetic results, and that this information would be additive (ie, scores would be higher for ε4 homozygous participants receiving an elevated amyloid result compared with groups receiving just 1 of these results). To assess outcomes controlling for potential covariate imbalance between these groups, we performed analysis of covariance (ANCOVA) models with amyloid group (elevated, intermediate, and not detected), *APOE* genotype (noncarrier, heterozygote, or homozygote), and amyloid group × *APOE* genotype interaction as independent variables, adjusting for age, sex, screening visit 1 scores on the Cognitive Function Index (CFI, a 15-item measure of concerns about memory performance), family history of disease, and study partner type (spouse vs nonspouse).

All statistical tests were 2-sided with a significance level prespecified at .05. No adjustments for multiple comparisons were made. Analyses were performed in R version 4.4.1 (R Project for Statistical Computing).

## Results

Table 1 describes the sample of participants from whom data were used in this study. Data were included for 3414 participants (mean [SD] age, 68.8 [6.0] years; 2116 [62%] were female; and 279 [8%] were Asian, 103 [3%] were Black, and 2971 [87%] were White). Among included participants, 1748 (51%) had not-detected amyloid, 482 (14%) had intermediate amyloid, and 1184 (35%) had elevated amyloid; 1468 (43%) were *APOE* ε4 noncarriers, 1609 (47%) were ε4 heterozygotes, and 337 (10%) were ε4 homozygotes. Individuals with elevated amyloid were slightly older than their intermediate and not detected counterparts; *APOE* ε4 heterozygotes and homozygotes were younger than their noncarrier counterparts within the amyloid groups. Among participants included in these analyses, data were missing for 310 participants for the IES and for 112 participants for the scale assessing concerns about AD dementia. Concerns about AD dementia before amyloid PET imaging were slightly higher with increasing amyloid level (elevated greater than intermediate, intermediate greater than not detected) and *APOE* gene dose (homozygotes greater than heterozygotes, heterozygotes greater than noncarrriers).

**Table 1.** Descriptive Summaries of the Study Participants

Characteristic	Participants by amyloid and *APOE* status, No. (%)									
	Not detected			Intermediate			Elevated			Total
	Noncarriers	Heterozygotes	Homozygotes	Noncarriers	Heterozygotes	Homozygotes	Noncarriers	Heterozygotes	Homozygotes	
Age, mean (SD)	68.5(5.9)	66.8 (6.2)	63.1 (5.3)	71.6 (4.5)	68.1 (5.4)	64.7 (5.2)	73.2 (4.6)	70.5 (5.2)	67.3 (5.6)	68.8 (6.0)
Sex										
Female	578 (57)	395 (61)	54 (58)	82 (59)	188 (70)	53 (72)	210 (66)	450 (65)	106 (62)	2116 (62)
Male	432 (43)	249 (39)	39 (42)	57 (41)	81 (30)	21 (28)	109 (34)	245 (35)	64 (38)	1297 (38)
Unknown	0	1 (<1)	0	0	0	0	0	0	0	1 (<1)
Race										
American Indian or Alaska Native	2 (<1)	2 (<1)	0	0	0	0	1 (<1)	1 (<1)	0	6 (<1)
Asian	158 (16)	52 (8)	2 (2)	11 (8)	16 (6)	2 (3)	22 (7)	13 (2)	3 (2)	279 (8)
Black or African American	38 (4)	20 (3)	3 (3)	9 (7)	9 (3)	1 (1)	7 (2)	12 (2)	4 (2)	103 (3)
Multiple	13 (1)	10 (2)	0	0	1 (<1)	0	2 (<1)	0	1 (<1)	27 (<1)
Native Hawaiian or Other Pacific Islander	0	0	0	0	0	0	0	1 (<1)	0	1 (<1)
Other^a^	8 (<1)	5 (<1)	0	3 (2)	1 (<1)	0	2 (<1)	4 (<1)	1 (<1)	24 (<1)
Unknown/not reported	1 (<1)	1 (<1)	0	0	0	0	0	0	1 (<1)	3 (<1)
White	790 (78)	555 (86)	88 (95)	116 (84)	242 (90)	71 (96)	285 (90)	664 (96)	160 (94)	2971 (87)
Ethnicity										
Hispanic	159 (16)	56 (9)	6 (7)	22 (16)	24 (9)	1 (1)	35 (11)	48 (7)	7 (4)	358 (11)
Not Hispanic	851 (84)	589 (91)	87 (94)	117 (84)	245 (91)	73 (99)	284 (89)	647 (93)	163 (96)	3056 (90)
Family history	717 (73)	515 (82)	74 (83)	89 (65)	225 (86)	65 (88)	227 (73)	569 (84)	153 (92)	2634 (80)
Study partner type										
Adult child	103 (10)	84 (13)	12 (13)	21 (13)	42 (16)	11 (15)	50 (16)	90 (13)	21 (12)	434 (13)
Other	299 (30)	181 (28)	17 (18)	44 (18)	64 (24)	21 (28)	87 (27)	179 (26)	28 (17)	920 (27)
Spouse	608 (60)	380 (59)	64 (69)	74 (69)	162 (60)	42 (57)	182 (57)	426 (61)	121 (71)	2059 (60)
Missing	0	0	0	0	1 (<1)	0	0	0	0	1 (<1)
CFI, mean (SD)	1.9 (2.2)	2.0 (2.2)	1.3 (1.8)	2.0 (2.2)	1.8 (2.0)	1.8 (2.5)	2.1 (2.1)	2.0 (2.1)	1.9 (1.9)	1.9 (2.1)
Concerns about AD, mean (SD)	21.0 (4.6)	22.2 (4.2)	22.4 (4.0)	21.2 (4.5)	22.0 (4.7)	23.1(3.9)	21.4 (4.9)	22.7 (4.4)	23.2 (3.9)	21.9 (4.5)

Abbreviations: AD, Alzheimer disease; *APOE*, apolipoprotein E; CFI, Cognitive Function Index.

^a^ Other includes Middle Eastern, Jewish, and other participant-presented responses.

Learning amyloid biomarker and *APOE* information was associated with measured intrusive thoughts 24 to 72 hours after disclosure (Table 2, Figure). Across genetics groups, learning an elevated amyloid result was associated with higher IES (mean [SD], 10.5 [10.9]) than intermediate amyloid (8.8 [9.8]), and learning an intermediate amyloid result was associated with higher scores than not-detected amyloid (6.5 [8.4]). Similarly, across amyloid groups, learning *APOE* ε4 homozygous status was associated with higher mean (SD) IES scores (12.7 [11.6]) than heterozygous status (9.1 [10.2]), and learning heterozygous status was associated with higher scores than noncarrier status (6.2 [8.1]). Table 2 provides the results for the 9 groups. In an ANCOVA model (Table 3), amyloid group (*F*_2_ = 31.9; *P* < .001) and *APOE* status (*F*_2_ = 28.7; *P* < .001) were significantly associated with IES score, although no interaction effect was observed for amyloid × *APOE*. Other covariates significantly associated with higher IES score included younger age, female sex, higher baseline CFI, and family history of AD.

**Table 2.** Raw Data Results for Impact of Events Scale (IES) and Concerns Alzheimer Disease (AD) Across Groups^a^

	Participants by amyloid and *APOE* status, mean (SD)									
	Not detected			Intermediate			Elevated			Total
	Noncarriers	Heterozygotes	Homozygotes	Noncarriers	Heterozygotes	Homozygotes	Noncarriers	Heterozygotes	Homozygotes	
IES	5.5 (7.6)	7.6 (9.1)	9.1 (10.0)	4.9 (6.3)	9.3 (10.1)	13.9 (11.0)	8.7 (9.8)	10.4 (10.9)	14.1 (12.4)	8.3 (9.8)
Change in concerns about AD	−1.2 (4.4)	−1.0 (4.0)	−0.5 (3.9)	0.6 (3.7)	0.4 (3.6)	0.5 (4.1)	0.9 (3.7)	0.8 (3.5)	0.9 (3.4)	−0.21 (4.0)

Abbreviation: *APOE*, apolipoprotein E.

^a^ Sample size in this table is smaller than Table 1; only participants undergoing screening visit 3 are included.

**Figure.**
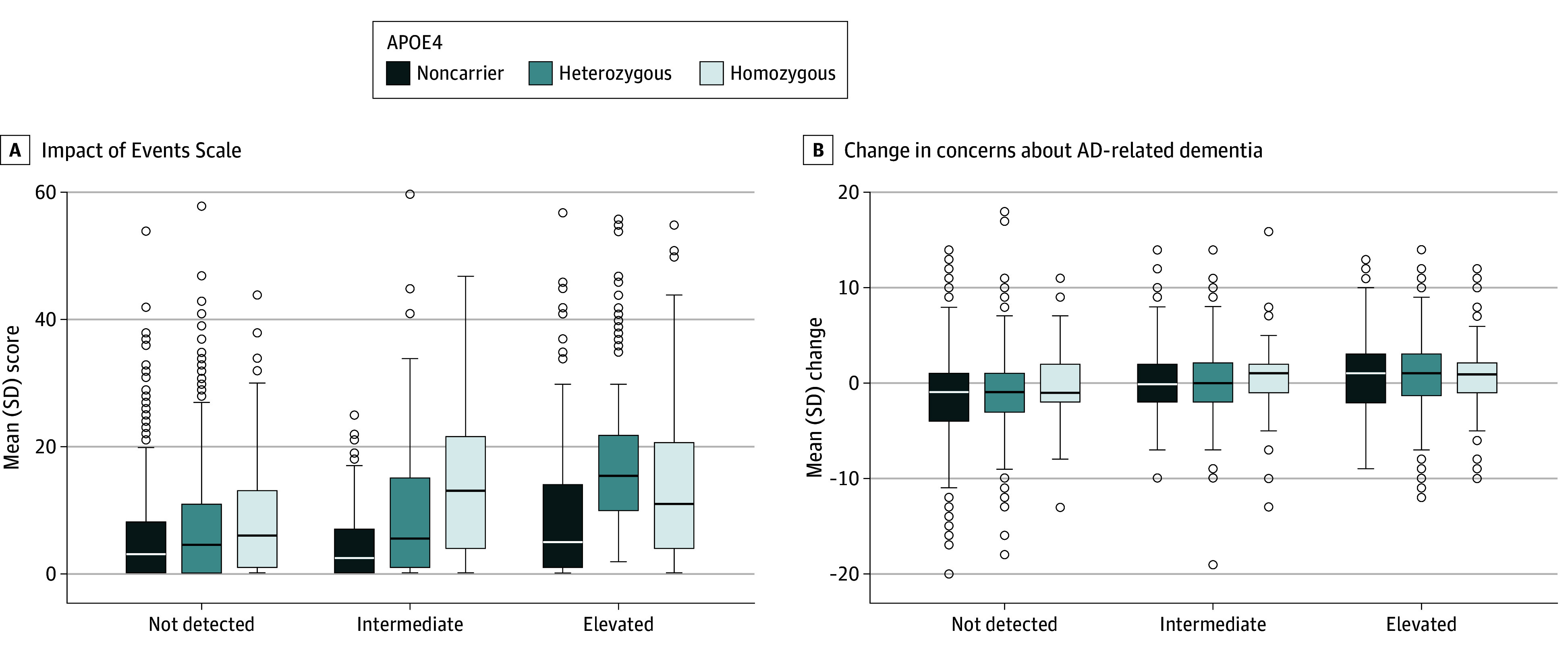
Box Plot of Group Scores for the Impact of Events Scale and Change in a Scale Measuring Concerns About Alzheimer Disease (AD) Dementia Among Amyloid Biomarker and Apolipoprotein E (*APOE*) Groups Dose associations of both types of information were observed for the Impact of Events Scale, whereas amyloid biomarker information but not *APOE* genotypes demonstrated similar effects for change in concerns about AD dementia. Boxes indicate IQR, lines indicate median, whiskers indicate the minimum and maximum excluding outliers, and circles indicate outliers.

**Table 3.** Outcomes of Analysis of Covariance Models

Measure	IES		Change in concerns about AD	
	*F* value	*P* value	*F* value	*P* value
PET	31.9	<.001	70.9	<.001
*APOE*	28.7	<.001	1.2	.31
Age	14.9	<.001	0.6	.42
Sex	65.5	<.001	0.00	.96
Cognitive Function Index	56.4	<.001	19.0	<.001
Family history	7.2	<.01	13.6	<.001
Study partner type	1.7	.18	0.6	.55
PET × *APOE*	1.8	.12	0.62	.65

Abbreviations: *APOE*, apolipoprotein E; PET, positron emission tomography.

Table 2 also presents the change in measured concerns about AD dementia among the 9 biomarker and genotype groups. There was a clear pattern of differential change in the level of concerns among the amyloid groups. Those with elevated amyloid showed a mean (SD) increase in concerns (0.8 [3.5]), those with intermediate amyloid showed a smaller increase (0.4 [3.7]), and those with not-detected amyloid showed a decrease in concerns (−1.1 [4.2]). Although groups based on *APOE* genotypes showed a similar pattern, the magnitude of mean (SD) changes and the differences between groups were smaller (ε4 homozygotes: 0.43 [3.7]; ε4 heterozygotes: 0.0 [3.8]; noncarriers: −0.6 [4.3]), and *APOE* was not associated with change in concerns in an ANCOVA model (*F* = 1.2; *P* = .31) (Table 3). In contrast, the amyloid PET group was significantly associated with change in concerns in the ANCOVA model (*F* = 70.9; *P* < .001). Other significant covariates in the ANCOVA model included screening CFI and family history of disease but not the interaction between amyloid group and *APOE* genotype.

## Discussion

To our knowledge, this is the first study to disclose 3 categories of amyloid biomarker results with differential information about potential risk of future decline to cognitively unimpaired participants. It is also among the first examinations, within a single study, of the outcomes of learning biomarker vs genetic information related to AD. Although results were collected in a clinical trial, which limits the generalizability of the findings, they can inform a clinical practice in which adults undergo biomarker testing and if biomarker positive, begin treatment.^18^ If this or other ongoing preclinical AD trials of AAMAs are positive, this new practice may include not only biomarker testing, but also genetic testing to inform patients of their treatment-related risks.^19^

Disclosure of biomarker and *APOE* results both were associated with intrusive thoughts and avoidance for cognitively unimpaired participants. We observed that the IES scores for *APOE* ε4 noncarriers receiving elevated amyloid results, on average, were about the same as those receiving a not-detected amyloid result who also learned they were ε4 homozygotes. As we hypothesized, the highest IES scores were observed among ε4 homozygotes receiving an elevated amyloid result (Table 2), although no formal statistical interaction was observed in an ANCOVA model. Somewhat surprisingly, the lowest group mean IES was observed for noncarriers receiving an intermediate amyloid result, although similar scores were observed among noncarriers receiving a not-detected result. The observed differences between the groups were relatively small, and none of the subgroup mean scores were in the range that would be considered clinically significant.^13^ In fact, the scores observed here align with previous studies,^20^ which found similar IES scores after disclosure of positive biomarker results,^11,15,21,22^ *APOE* ε4 carrier status,^23,24,25^ and carriage of autosomal dominant AD gene variants.^14^ Together, the results further support that information about risk of impending cognitive decline and dementia can be delivered safely to unimpaired individuals who receive education before testing.

Remarkably, and aligned with previous results,^11,26^ the amyloid and *APOE* groups differed at baseline (before testing and disclosure) in their level of concerns. The results also align with previous studies about the specific subgroups who may be at risk for greater distress related to receiving this information, including females, older adults of younger ages, individuals with a family history of dementia, and those who experience and express concerns about changes in cognitive performance.^11^

Even though genetic and biomarker results can safely be delivered, they have may have impact that needs to be considered before testing and disclosure. Information about the brain is sensitive and seen as unique to other medical test results.^27^ Learning about an intermediate or elevated amyloid result was associated with statistically significantly greater increases in concerns about AD dementia compared with learning a not-detected result. A not-detected result was associated with reduced concerns about AD dementia. No differences in change in concerns about AD dementia were observed based on genetic status for the elevated or intermediate amyloid groups, suggesting that participants prioritized biomarker over genetic information when considering their future risk. The exception to this was in the not-detected amyloid group; noncarriers seemed to experience the greatest reduction in concerns, while homozygotes experienced a smaller reduction.

### Limitations

Several limitations of this study should be noted. Some individuals opted out of learning *APOE* genotype and some had delayed access to *APOE* results and were therefore not included in the current analyses. Our data do not permit distinction of these unique groups, and our exclusion of both groups introduces the potential for selection bias. It is possible that some participants already knew their amyloid biomarker results, for example through participation in other studies, or their *APOE* results, for example through direct-to-consumer testing. Learning the amyloid PET result was framed as the event in the IES in this study. This may have muted or otherwise affected data on this scale for *APOE* compared with amyloid PET disclosure, although the observations here suggest that *APOE* disclosure was independently associated with IES. Concerns about AD dementia were assessed with an adapted scale immediately after the disclosure while IES was collected via phone 24 to 72 hours after disclosure, caveats to comparisons between the outcomes. Precision was lower for outcomes in the intermediate amyloid group due to the smaller sample size, and this was particularly true for subgroups based on *APOE* in this amyloid category. Due to limited inclusion and very small sample sizes within groups, these analyses did not assess for differences based on race, ethnicity, or social determinants of health. Midstudy, plasma biomarker testing was introduced and used to enrich the sample advancing to amyloid imaging. We analyzed data from a mixed population that included some people who had their baseline concerns about AD dementia collected after they were told they were preliminarily eligible to proceed with screening based on plasma testing. In sensitivity analyses, comparing groups based on whether they were enrolled before or after the implementation of plasma biomarker enrichment did not affect the main observations reported here (data not shown).

## Conclusions

In conclusion, this study provides novel information about psychological outcomes after disclosure of AD biomarker and *APOE* genetic information in cognitively unimpaired adults. While both types of information have statistically significant associations with intrusive thoughts, only biomarker results were observed to be associated with adjusted perceived risk of AD dementia.

## Notes

Supplement 1. eTable. Descriptive Summaries of the Included vs the not Included AHEAD Participants Undergoing Amyloid PET

Supplement 2. Data Sharing Statement

## References

[1] Jack CR Jr, Andrews JS, Beach TG, . Revised criteria for diagnosis and staging of Alzheimer’s disease: Alzheimer’s Association Workgroup. Alzheimers Dement. 2024;20(8):5143-5169. doi:10.1002/alz.13859 38934362 PMC11350039

[2] Dubois B, Villain N, Schneider L, . Alzheimer disease as a clinical-biological construct-an international working group recommendation. JAMA Neurol. 2024;81(12):1304-1311. doi:10.1001/jamaneurol.2024.3770 39483064 PMC12010406

[3] Boxer AL, Sperling R. Accelerating Alzheimer’s therapeutic development: the past and future of clinical trials. Cell. 2023;186(22):4757-4772. doi:10.1016/j.cell.2023.09.023 37848035 PMC10625460

[4] Rafii MS, Sperling RA, Donohue MC, . The AHEAD 3-45 study: design of a prevention trial for Alzheimer’s disease. Alzheimers Dement. 2022;•••. doi:10.1002/alz.12748 35971310 PMC9929028

[5] Bullich S, Roé-Vellvé N, Marquié M, . Early detection of amyloid load using ^18^F-florbetaben PET. Alzheimers Res Ther. 2021;13(1):67. doi:10.1186/s13195-021-00807-6 33773598 PMC8005243

[6] Landau SM, Horng A, Jagust WJ; Alzheimer’s Disease Neuroimaging Initiative. Memory decline accompanies subthreshold amyloid accumulation. Neurology. 2018;90(17):e1452-e1460. doi:10.1212/WNL.0000000000005354 29572282 PMC5921038

[7] Sims JR, Zimmer JA, Evans CD, ; TRAILBLAZER-ALZ 2 Investigators. Donanemab in early symptomatic Alzheimer disease: the TRAILBLAZER-ALZ 2 randomized clinical trial. JAMA. 2023;330(6):512-527. doi:10.1001/jama.2023.13239 37459141 PMC10352931

[8] van Dyck CH, Swanson CJ, Aisen P, . Lecanemab in early Alzheimer’s disease. N Engl J Med. 2022;388(1):9-21. doi:10.1056/NEJMoa2212948 36449413

[9] Zimmer JA, Ardayfio P, Wang H, . Amyloid-related imaging abnormalities with donanemab in early symptomatic Alzheimer disease: secondary analysis of the TRAILBLAZER-ALZ and ALZ 2 randomized clinical trials. JAMA Neurol. 2025;82(5):461-469. doi:10.1001/jamaneurol.2025.0065 40063015 PMC11894547

[10] Harkins K, Sankar P, Sperling R, . Development of a process to disclose amyloid imaging results to cognitively normal older adult research participants. Alzheimers Res Ther. 2015;7(1):26. doi:10.1186/s13195-015-0112-7 25969699 PMC4428104

[11] Grill JD, Raman R, Ernstrom K, ; A4 Study Team. Short-term psychological outcomes of disclosing amyloid imaging results to research participants who do not have cognitive impairment. JAMA Neurol. 2020;77(12):1504-1513. doi:10.1001/jamaneurol.2020.2734 32777010 PMC7418046

[12] Rissman RA, Langford O, Raman R, . Plasma Abeta42/Abeta40 and phospho-tau217 concentration ratios increase the accuracy of amyloid PET classification in preclinical Alzheimer’s disease. Alzheimers Dement. 2023;20(2):1214-1224. doi:10.1002/alz.13542 37932961 PMC10916957

[13] Horowitz M, Wilner N, Alvarez W. Impact of event scale: a measure of subjective stress. Psychosom Med. 1979;41(3):209-218. doi:10.1097/00006842-197905000-00004 472086

[14] Cassidy MR, Roberts JS, Bird TD, . Comparing test-specific distress of susceptibility versus deterministic genetic testing for Alzheimer’s disease. Alzheimers Dement. 2008;4(6):406-413. doi:10.1016/j.jalz.2008.04.007 19012865 PMC2610442

[15] Caprioglio C, Ribaldi F, Visser LNC, ; AMYPAD consortium. Analysis of psychological symptoms following disclosure of amyloid-positron emission tomography imaging results to adults with subjective cognitive decline. JAMA Netw Open. 2023;6(1):e2250921. doi:10.1001/jamanetworkopen.2022.50921 36637820 PMC9857261

[16] Roberts JS, Connell CM. Illness representations among first-degree relatives of people with Alzheimer disease. Alzheimer Dis Assoc Disord. 2000;14(3):129-136. doi:10.1097/00002093-200007000-00003 10994653

[17] Ashida S, Koehly LM, Roberts JS, Chen CA, Hiraki S, Green RC. The role of disease perceptions and results sharing in psychological adaptation after genetic susceptibility testing: the REVEAL Study. Eur J Hum Genet. 2010;18(12):1296-1301. doi:10.1038/ejhg.2010.119 20664629 PMC2988099

[18] Sperling RA, Karlawish J, Johnson KA. Preclinical Alzheimer disease-the challenges ahead. Nat Rev Neurol. 2013;9(1):54-58. doi:10.1038/nrneurol.2012.241 23183885 PMC3643203

[19] Knopman DS, Hershey L. Implications of the approval of lecanemab for Alzheimer disease patient care: incremental step or paradigm shift? Neurology. 2023;101(14):610-620. doi:10.1212/WNL.0000000000207438 37295957 PMC10573150

[20] Bemelmans SA, Tromp K, Bunnik EM, . Psychological, behavioral and social effects of disclosing Alzheimer’s disease biomarkers to research participants: a systematic review. Alzheimers Res Ther. 2016;8(1):46. doi:10.1186/s13195-016-0212-z 27832826 PMC5103503

[21] Wake T, Tabuchi H, Funaki K, . The psychological impact of disclosing amyloid status to Japanese elderly: a preliminary study on asymptomatic patients with subjective cognitive decline. Int Psychogeriatr. 2018;30(5):635-639. doi:10.1017/S1041610217002204 29094656

[22] Clark LR, Erickson CM, Chin NA, . Psychosocial implications of learning amyloid PET results in an observational cohort. Alzheimers Dement. 2024;20(9):6579-6589. doi:10.1002/alz.14153 39129396 PMC11497643

[23] Green RC, Roberts JS, Cupples LA, . Disclosure of APOE genotype for risk of Alzheimer’s disease. N Engl J Med. 2009;361(3):245-54. doi:10.1056/NEJMoa0809578 PMC2778270 19605829

[24] Alber J, Popescu D, Thompson LI, . Safety and tolerability of APOE genotyping and disclosure in cognitively normal volunteers from the Butler Alzheimer’s prevention registry. J Geriatr Psychiatry Neurol. 2022;35(3):293-301. doi:10.1177/0891988721993575 33550928

[25] Christensen KD, Uhlmann WR, Roberts JS, . A randomized controlled trial of disclosing genetic risk information for Alzheimer disease via telephone. Genet Med. 2018;20(1):132-141. doi:10.1038/gim.2017.103 PMC5897910 28726810

[26] Grill JD, Raman R, Ernstrom K, . Immediate reactions to Alzheimer biomarker disclosure in cognitively unimpaired individuals in a global truncated randomized trial. Neurol Clin Pract. 2024;14(2):e200265. doi:10.1212/CPJ.0000000000200265 38585443 PMC10996909

[27] Largent EA, Harkins K, van Dyck CH, Hachey S, Sankar P, Karlawish J. Cognitively unimpaired adults’ reactions to disclosure of amyloid PET scan results. PLoS One. 2020;15(2):e0229137. doi:10.1371/journal.pone.0229137 32053667 PMC7018056

