# Supplementary material for: Amyloid Imaging and APOE Genotype Disclosure and Short-Term Psychological Distress
Source: JAMA Netw Open. 2026 Mar 30;9(3):e263845. doi: 10.1001/jamanetworkopen.2026.3845 (PMC13036580; doi:10.1001/jamanetworkopen.2026.3845)
Supplement: Supplement 1. — eTable. Descriptive Summaries of the Included vs the not Included AHEAD Participants Undergoing Amyloid PET [file jamanetwopen-e263845-s001.pdf]

Supplemental Online Content

Grill JD, Raman R, Wang S, et al. Amyloid imaging, APOE genotype disclosure, and short-term psychological distress. *JAMA Netw Open*. 2026;9(3):e263845.
doi:10.1001/jamanetworkopen.2026.3845

eTable. Descriptive Summaries of the Included vs the not Included AHEAD Participants Undergoing Amyloid PET

This supplemental material has been provided by the authors to give readers additional information about their work.

© 2026 Grill JD et al. *JAMA Network Open*.

eTable. Descriptive Summaries of the Included vs the not Included AHEAD Participants Undergoing Amyloid PET			
	Included<br>(N = 3414)	Not Included<br>(N = 1067)	Total<br>(N = 4481)
Age, mean (SD)	68.8 (6.0)	68.6 (6.0)	
Sex, n (%)			
Female	2116 (62%)	662 (62%)	2778 (62%)
Male	1297 (38%)	405 (38%)	1702 (38%)
Race, n (%)			
White	2971 (87%)	903 (85%)	3874 (87%)
American Indian or Alaska Native	6 (<1%)	2 (<1%)	8 (<1%)
Asian	279 (8%)	108 (10%)	387 (9%)
Black or African American	103 (3%)	29 (3%)	132 (3%)
Native Hawaiian or other Pacific Islander	1 (<1%)	2 (<1%)	3 (<1%)
Multiple	27 (1%)	14 (1%)	41 (1%)
Other	24 (1%)	7 (1%)	31 (1%)
Unknown/Not reported	3 (<1%)	2 (<1%)	5 (<1%)
Ethnicity, n (%)			
Hispanic	358 (11%)	91 (9%)	449 (10%)
Not Hispanic	3056 (89%)	976 (91%)	4032 (90%)
Family history	2634 (79%)	772 (75%)	3406 (78%)
Study partner type, n (%)			
Adult child	434 (13%)	128 (12%)	562 (13%)
Spouse	2059 (60%)	636 (28%)	2695 (60%)
Other	920 (27%)	301 (28%)	1221 (27%)
CFI, mean (SD)	1.9 (2.1)	2.0 (2.3)	2.0 (2.0)
Concerns about AD, mean (SD)	21.9 (4.5)	22.2 (4.4)	22.0 (4.5)

© 2026 Grill JD et al. *JAMA Network Open*.
