# Supplementary material for: Amyloid Imaging and APOE Genotype Disclosure and Short-Term Psychological Distress
Source: JAMA Netw Open. 2026 Mar 30;9(3):e263845. doi: 10.1001/jamanetworkopen.2026.3845 (PMC13036580; doi:10.1001/jamanetworkopen.2026.3845)
Supplement: Supplement 2. — Data Sharing Statement [file jamanetwopen-e263845-s002.pdf]

Data Sharing Statement

Data
Data available: Yes
Data types: Deidentified participant data
How to access data: Deidentified data from North American participants used in this publication, along with relevant data dictionary, will be made freely available to the public on October 15, 2025 through the AHEAD Study website (www.aheadstudydata.org).
When available: With publication

Supporting Documents
Document types: Statistical/analytic code
How to access documents: The website will include a data dictionary
When available: With publication

Additional Information
Who can access the data: Anyone requesting the data
Types of analyses: Any scientific purpose
Mechanisms of data availability: With or without support
Any additional restrictions: No additional restrictions
